# Impact of Azithromycin and/or Hydroxychloroquine on Hospital Mortality in COVID-19

**DOI:** 10.3390/jcm9092800

**Published:** 2020-08-30

**Authors:** Filippo Albani, Federica Fusina, Alessia Giovannini, Pierluigi Ferretti, Anna Granato, Chiara Prezioso, Danilo Divizia, Alessandra Sabaini, Marco Marri, Elena Malpetti, Giuseppe Natalini

**Affiliations:** 1 Department of Anesthesia, Intensive Care and Pain Medicine, Fondazione Poliambulanza Istituto Ospedaliero, 25124 Brescia, Italy; f.fusina@gmail.com (F.F.); alessia.giovannini@poliambulanza.it (A.G.); pierluigi.ferretti@poliambulanza.it (P.F.); anna.granato@poliambulanza.it (A.G.); c.prezioso89@gmail.com (C.P.); danilo.divizia@poliambulanza.it (D.D.); alessandra.sabaini@poliambulanza.it (A.S.); elena.malpetti@poliambulanza.it (E.M.); giuseppe.natalini@poliambulanza.it (G.N.); 2 Department of Intensive Care Medicine and Anaesthesiology, Fondazione Policlinico Universitario A. Gemelli, Università Cattolica del Sacro Cuore, 00168 Rome, Italy; 3 Department of Information and Communications Technology, Fondazione Poliambulanza Istituto Ospedaliero, 25124 Brescia, Italy; marco.marri@poliambulanza.it

**Keywords:** azithromycin, hydroxychloroquine, SARS-CoV-2

## Abstract

The severe acute respiratory syndrome coronavirus 2 (SARS-CoV-2) pandemic has led to widespread use of hydroxychloroquine and azithromycin despite the lack of conclusive evidence for their safety and efficacy. We evaluated the association between treatment with hydroxychloroquine and/or azithromycin and hospital mortality as the primary outcome. We compared the hospital mortality of patients treated with hydroxychloroquine alone, azithromycin alone, or their combination to the mortality of patients who received neither drug. A logistic multivariate model with overlap weight propensity score was used for estimation of odds ratios (ORs) with 95% confidence intervals (95% CIs). One thousand four hundred and three patients with SARS-CoV-2 infection were admitted to the hospital. At the time of the analysis, the outcome was available for 1376 (98%) of them. Five hundred and eighty-seven patients (42%) received azithromycin and 377 patients (27%) received hydroxychloroquine, alone or in combination. In-hospital mortality was 26%. After the adjusted analysis, azithromycin alone was associated with lower mortality (OR 0.60, 95% CI 0.42–0.85) compared to no treatment. Hydroxychloroquine alone (OR 0.76, 95% CI 0.53–1.08) and the combination of azithromycin and hydroxychloroquine (OR 1.13, 95% CI 0.77–1.69) were not associated with hospital mortality. In this cohort of patients, azithromycin alone was associated with lower hospital mortality but hydroxychloroquine was not associated with increased or reduced mortality. While we await randomized clinical trials, these data support the use of azithromycin in novel coronavirus disease 2019 (COVID-19) and can contribute to better understanding of its role in further meta-analyses.

## 1. Introduction

Novel coronavirus disease 2019 (COVID-19) has an impressively high mortality rate, even outside intensive care units (ICUs) [1]. Northern Italy was hit hard, and Lombardy, where half of Italy’s deaths occurred, was especially impacted [2]. Evidence concerning an effective pharmacological treatment is still inconclusive. In the absence of available evidence-based treatments, azithromycin and hydroxychloroquine have been proposed as drugs that could theoretically be effective in treating COVID-19 [3,4]. An open-label non-randomized clinical trial demonstrated the superiority of hydroxychloroquine alone or in combination with azithromycin in viral load reduction, suggesting a synergistic effect between the two drugs [5].

Based on this evidence, the compassionate use of hydroxychloroquine in combination with azithromycin for patients affected by severe acute respiratory syndrome coronavirus 2 (SARS-CoV-2) pneumonia has become common clinical practice [6]. Nonetheless, two large observational studies reported no association between treatment with hydroxychloroquine and/or azithromycin and mortality [7,8].

The debate is ongoing after a large observational study, which showed an increase in mortality in patients treated with hydroxychloroquine, was retracted [9]. The study’s results, however, had already led the World Health Organization to temporarily suspend enrollment in randomized clinical trials that used hydroxychloroquine.

Another recent clinical trial that randomized patients to hydroxychloroquine or placebo for post-exposure prophylaxis has shown no difference in the incidence of infection [10].

Gathering solid evidence is therefore crucial, especially because recurrence of infection in colder months for other human coronaviruses is common; hence, it is not possible to exclude a second surge of COVID-19 [11].

The aim of this study was to compare the effect of treatment with hydroxychloroquine, azithromycin, or their combination versus no therapy in patients admitted for SARS-CoV-2 infection.

## 2. Experimental Section

### 2.1. Study Sample

This study was conducted according to the Declaration of Helsinki and the protocol was approved by Brescia’s Ethical Committee (NP4218), which allowed a waiver of consent from individual patients due to the retrospective nature of the study. We included all patients admitted between 20 February 2020 and 10 May 2020 to Fondazione Poliambulanza, a tertiary referral hospital with 600 beds located in Brescia (Lombardy), who had a positive real-time polymerase chain reaction (RT-PCR) for SARS-CoV-2 from a nasal swab or other biological material. Demographic, clinical and laboratory data were prospectively collected from the electronic medical charts of the enrolled patients.

### 2.2. Exposure

Patients were divided in four groups based on the treatment they received during hospitalization: (1) neither drug, if patients did not receive hydroxychloroquine or azithromycin during their hospital stay; (2) hydroxychloroquine without azithromycin; (3) azithromycin without hydroxychloroquine; and (4) hydroxychloroquine with azithromycin. Dosing regimens in our institution were 500 mg per day for 5 days for azithromycin and 200 mg bid for 5–7 days for hydroxychloroquine, based on the judgment of the treating physician.

### 2.3. Outcomes

The primary outcome was hospital mortality. Admission to the ICU and hospital length of stay were the secondary outcomes.

### 2.4. Statistical Analysis

We expected to collect complete information on 1400 patients. With an overall mortality of 25%, this guaranteed a sufficient sample size to investigate all recorded variables in the multivariate model [12]. Seven hundred and eighty-eight patients were needed to obtain a power of 80% to detect an odds ratio (OR) of 0.65, assuming mortality was 25% in those receiving hydroxychloroquine, and expecting that one patient would receive hydroxychloroquine for every three that would not, with alpha = 0.05 [13]. The 12-week follow-up ensured that 97% of admitted patients had been discharged or had died by the end of the study period. Continuous variables were described with the median (1st and 3rd quartiles), and factor variables with frequency (percentage). Univariate analysis of the outcome was performed on variables selected a priori, using Fisher’s test for factorial variables and the Kruskal–Wallis test for continuous ones. Bivariate association with treatments was assessed. A propensity score for treatment allocation was estimated from a multivariable logistic regression model containing patient age, sex, ratio of partial pressure of arterial oxygen to fraction of inspired oxygen (PaO_2_/FiO_2_), lactate, C-reactive protein, ICU admission, and platelet count. The overlap weight propensity score method was then applied to a multivariate logit regression modeling the primary outcome (in-hospital mortality), and ORs with 95% confidence intervals (95% CIs) are reported. Collinearity was assessed with variance inflation factor (VIF) and variables excluded from the multivariate model if VIF was greater than 3 and there was correlation with other explanatory variables (e.g., PaO_2_/FiO_2_ and PaO_2_) [14]. The same procedure was used for ORs of ICU admission. The overlap weight method was used with a Poisson multivariate analysis to model in-hospital length of stay and incidence rate ratios (IRRs) (95% CIs) were reported [15]. No treatment was compared to azithromycin alone, hydroxychloroquine alone, or to the combination of hydroxychloroquine and azithromycin. If a statistically significant association was found, we computed the E-values for the point estimate and the CI limit closer to the null, in order to assess the possible effect of unmeasured confounders [16]. Missing values were imputed with mean substitution. Two sensitivity analyses were planned: (1) excluding patients admitted to ICU and (2) analysis of complete cases. An additional sensitivity analysis was conducted after the peer-review process, updating the results with the inclusion of patients admitted to hospital from 10 May 2020 to 19 August 2020. The analysis was conducted in R version 4.0.0 (R Core Team, 2014), package “psw” for overlap weight propensity score and package “Evalue”. Significance was evaluated at α  = 0.05 and all testing was two-sided.

## 3. Results

**Figure 1:**
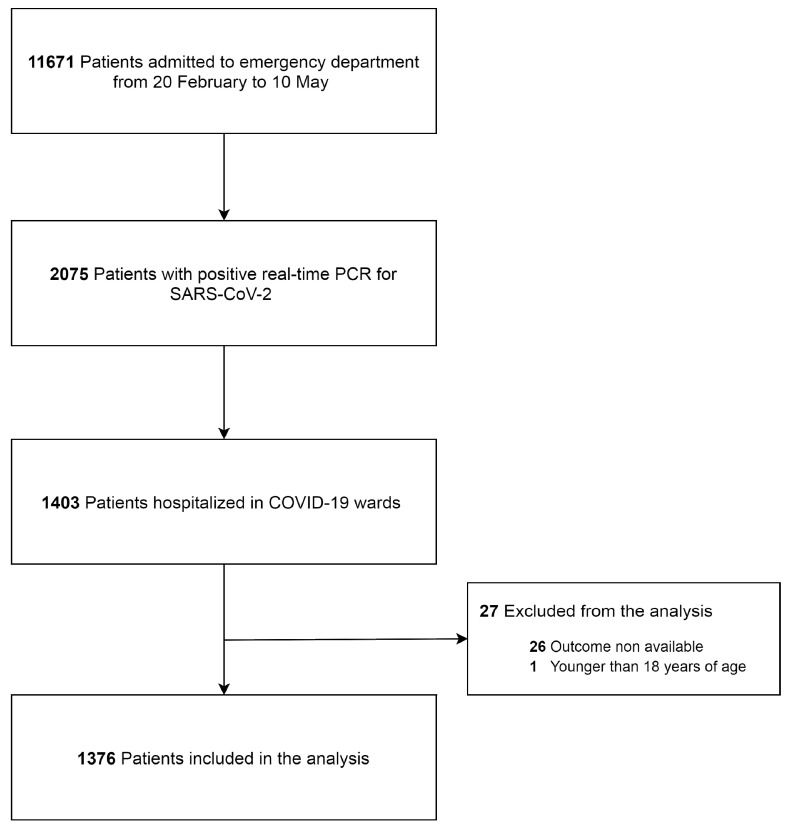
Severe acute respiratory syndrome coronavirus 2 (SARS-CoV 2). Coronavirus disease (COVID-19).

Between 20 February and 10 May 2020, 1403 patients with SARS-CoV-2 infection were admitted to the hospital (Figure 1). The distribution of patients’ characteristics in the four treatment groups is displayed in Table 1. The analysis was conducted on 1376 (98%) patients, for whom outcome was available at the time of analysis. One hundred and sixty-six (12%) patients received the combination of hydroxychloroquine and azithromycin, 421 (30%) azithromycin alone, 211 (15%) hydroxychloroquine alone, and 605 (43%) neither treatment. The median age of admitted patients was 70 (18) years, and the majority of them were male (924; 66%). At hospital admission, patients were hypoxemic and hypocapnic. Univariate association with hospital mortality is shown in Table 2. Deceased patients were older than survivors and more frequently male. A history of hypertension or diabetes was not associated with hospital mortality. Deceased patients were significantly more hypoxemic and hypocapnic than patients who did not die. Serum lactate, C-reactive protein, and lactate dehydrogenase at hospital admission were lower in survivors. Thrombocytopenia and lymphocytopenia were significantly associated with an adverse outcome. Patients admitted to intensive care were more likely to die during hospitalization. Four hundred and twenty-one (30%) patients were treated with azithromycin alone, their median age was 71 years old, and 62.2% of them were male. The prevalence of arterial hypertension and diabetes mellitus were not different when compared with the other groups (*p* = 0.87 and 0.23, respectively). Patients’ and laboratory characteristics are displayed in Table 1. Sixty-nine (16.4%) patients treated with azithromycin alone died during hospitalization.

### 3.1. Primary Outcome

In-hospital mortality was 26%, and it was significantly different between the four groups: it was 28% in patients who did not receive treatment with either azithromycin or hydroxychloroquine, 16% in those who received azithromycin alone, 28% in those who received only hydroxychloroquine, and 32% in patients who were treated with both drugs. In the unadjusted analysis, azithromycin versus neither treatment was significantly associated with reduced mortality (OR 0.48, 95% CI 0.35–0.65). Hydroxychloroquine versus neither treatment (OR 0.98, 95% CI 0.69–1.38), and azithromycin and hydroxychloroquine versus neither treatment (OR 1.15, 95% CI 0.79–1.66) were not associated with mortality in the unadjusted analysis. Variables included in the multivariate model were age, sex, admission to intensive care, PaO_2_/FiO_2_, C-reactive protein, serum lactate, platelets, and treatment with corticosteroids and/or enoxaparin. Overlap weight propensity score was able to resolve the difference between treatment groups in the examined variables (Figures S1–S3, Supplementary Materials). Estimates of ORs obtained with the overlap weight propensity score are reported in Table 3. Azithromycin alone was associated with lower mortality (OR 0.60, 95% CI 0.42–0.85) compared with no treatment, E-value 1.90 for the estimated OR, and E-value 1.38 for the upper CI limit. Hydroxychloroquine alone (OR 0.76, 95% CI 0.53–1.09) and the combination of azithromycin and hydroxychloroquine (OR 1.13, 95% CI 0.77–1.69) were not associated with hospital mortality. Sensitivity analyses yielded similar results for the primary outcome. Since 10 May, 74 other patients have been admitted to hospital for COVID-19. The inclusion of these patients in the analysis resulted in a similar estimate for OR to that previously provided---that is, treatment with azithromycin alone (OR 0.64, 95% CI 0.45–0.90), hydroxychloroquine alone (OR 0.87, 95% CI 0.61–1.22), and the combination of azithromycin and hydroxychloroquine (OR 1.28, 95% CI 0.88–1.88).

### 3.2. Secondary Outcomes

Results of unadjusted and adjusted analyses on ICU admission were in agreement. Treatment with azithromycin alone (OR 1.08, 95% CI 0.57–2.05) or hydroxychloroquine alone (OR 1.10, 95% CI 0.69–1.76) was not associated with ICU admission compared with neither treatment. Patients receiving azithromycin and hydroxychloroquine had a greater risk of being admitted to the ICU, according to the adjusted analysis (OR 1.82, 95% CI 1.27–2.61), E-value 2.03 for the estimated OR, and E-value 1.50 for the lower CI. Hospital length of stay was significantly longer for patients in all treatment groups compared with neither treatment (in both unadjusted and adjusted analyses). In particular, azithromycin alone had an IRR of 1.17 (95% CI 1.10–1.25), hydroxychloroquine alone had an IRR of 1.15 (95% CI 1.06–1.24), and azithromycin and hydroxychloroquine had an IRR of 1.34 (95% CI 1.24–1.45).

## 4. Discussion

We report a protective effect of azithromycin on the primary outcome of in-hospital mortality.

Our cohort of patients had similar characteristics to those reported in other studies [2,7]. In addition, the association of patients’ characteristics with mortality was similar to that previously reported: male sex, age, and lymphopenia were independently associated with a negative outcome at hospital discharge [7,8,16]. On the contrary, a history of arterial hypertension or diabetes mellitus was not associated with death. Patients’ characteristics were balanced with the propensity score of the measured variables, ensuring that treatment groups were comparable. This was aimed at limiting the effect of confounders on the outcome [17] (see Supplementary Materials). After balancing for variables associated with mortality, azithromycin maintained a protective effect on hospital mortality. Azithromycin has been shown to have a direct antiviral effect on rhinovirus replication, by enhancing the expression of interferon (IFN) and IFN-stimulated genes (ISGs) in human bronchial epithelial cells. This could hypothetically explain the inhibition of SARS-CoV-2 replication [18]. Its antiviral activity has been documented in vitro for Zika and Ebola viruses [19,20]. To our knowledge, this is the first observational study showing a significant effect of azithromycin on reducing mortality in COVID-19 patients. Data from Rosenberg et al. [7] show a trend, although not statistically significant, towards mortality reduction in patients treated with azithromycin compared to patients not receiving the treatment. This difference may have been due to the number of patients treated with azithromycin in the two studies, since we had twice as many patients who received azithromycin (211 vs. 421). In our patients, no effect on in-hospital mortality was highlighted by the use of hydroxychloroquine alone or in combination with azithromycin. This result agrees with the reports by Geleris et al. [8] and Rosenberg et al. [7] and supports the recommendation that hydroxychloroquine should not be routinely prescribed in patients with SARS-CoV-2 pneumonia. Moreover, a randomized clinical trial, which had to be prematurely stopped, documented that hydroxychloroquine in high doses increased the risk of arrhythmogenic cardiac death [21]. This evidence is in agreement with data indicating that the combination of azithromycin and hydroxychloroquine may lengthen QTc and predispose to arrhythmias [22]. This harmful side effect could increase mortality in the group of patients treated with the combination of azithromycin and hydroxychloroquine, and thus cancel the observed protective effect of azithromycin.

In our study, patients who received hydroxychloroquine and/or azithromycin had a longer hospital stay than patients who did not receive either drug. This might be in part related to the fact that survivors had a longer length of stay than non-survivors. The length of hospitalization in our patient cohort was generally longer than that reported by Rosenberg et al., which could have been due to different criteria for hospital discharge. Nevertheless, our patients were older than the ones in Rosenberg’s cohort, and therefore likely needed longer hospitalization. The intensive care admission rate was higher in patients receiving hydroxychloroquine with azithromycin compared to patients receiving standard care. These findings are in line with the reports from New York City, although the overall admission rate was lower in our cohort. The saturation of ICU capacity during the epidemic crisis could have played a role in the lower rate of ICU admission [2]. We chose to use a logistic model in the analysis instead of a Cox model because, as shown by the survival curve reported by Rosenberg et al., hospital mortality is definite after 2 weeks. We also did not intend to analyze the course of survival over time, but rather we were interested in the outcome of the disease at hospital discharge.

The limitations of this study are related to its observational nature; therefore, it cannot be excluded that the reported effect was the result of some unmeasured confounder or bias. This requires further research and randomized clinical trials to minimize the risk of bias. Similar to previous records, the dosage of hydroxychloroquine or azithromycin and the timing of therapy initiation related to symptoms onset were variables that we chose not to include in our analysis. Nonetheless, our data can be taken into consideration for future meta-analyses, along with data from similar studies. They can also help with the interpretation of the results of future randomized clinical trials, which does not always appear univocal and exhaustive.

## 5. Conclusions

This study supports the idea that azithromycin should be considered as a treatment for COVID-19, since it was effective in reducing in-hospital mortality. No benefit was shown for patients treated with hydroxychloroquine.

## Figures and Tables

**Table 1:** Patients’ characteristics and outcome in the four groups.

		Neither Ttreatment	HCQ Aalone	AZT Aalone	AZT and HCQ	*p*-Value
Patients	N (%)	605 (43)	211 (15)	421 (30)	166 (12)	
Age	years	72 (60–81)	68 (59–74)	71 (59–79)	70 (62–75)	<0.001
Body mass index	kg∗m^−2^	26 (23–29)	26 (24–29)	26 (23–29)	26 (24–29)	0.007
Male	N (%)	387 (64.0)	155 (73.5)	262 (62.2)	120 (72.3)	0.008
**Comorbidity**						
Arterial hypertension	N (%)	210 (34.7)	73 (34.6)	156 (37.1)	60 (36.1)	0.871
Diabetes mellitus	N (%)	137 (22.6)	36 (17.1)	79 (18.8)	37 (22.3)	0.229
**Laboratory data**						
PaO_2_	mmHg	54 (45–65)	52 (42–64)	57 (48–68)	53 (45–60)	0.009
PaCO_2_	mmHg	34 (30–38)	34 (31–38)	34 (31–38)	33 (31–37)	0.359
PaO_2_/FiO_2_	mmHg	254 (204–299)	237 (180–286)	274 (226–323)	257 (217–288)	<0.001
pH		7.48 (7.44–7.51)	7.49 (7.46–7.52)	74.8 (7.46–7.51)	7.49 (7.47–7.52)	<0.001
Lactate	Mmol∗L^–1^	1.1 (0.8–1.7)	1.0 (0.8–1.4)	1.0 (0.7–1.4)	1.0 (0.8–1.4)	<0.001
Leukocytes	10^9^∗L^−1^	7.8 (5.9–10.6)	7.1 (5.3–9.9)	7.0 (5.2–9.2)	7.0 (5.3–9.4)	0.006
Lymphocytes	10^9^∗L^−1^	0.9 (0.6–1.3)	0.9 (0.6–1.2)	1.0 (0.7–1.4)	0.9 (0.7–1.2)	0.002
Platelets	10^9^∗L^−1^	195 (144–255)	171 (132–243)	176 (136–230)	164 (132–231)	0.002
C-reactive protein	mg∗L^−1^	106 (41–177)	140 (73–198)	74 (36–142)	108 (62–167)	<0.001
Lactate dehydrogenase	U∗L^−1^	433 (304–578)	642 (506–794)	470 (334–567)	417 (319–529)	0.042
**Outcomes**						
In-hospital mortality	N (%)	172 (28.4)	60 (28.4)	69 (16.4)	53 (31.9)	<0.001
ICU admission	N (%)	46 (7.6)	73 (34.6)	20 (4.8)	48 (28.9)	<0.001
Hospital length of stay	days	6 (4–9)	10 (6–16)	6 (4–10)	10 (7–18)	<0.001

Demographics, comorbidities, laboratory data, and outcomes of the study subjects. Factor variables are expressed as count (%), continuous variables as median (IQR). All comparisons were statistically significant, except for arterial hypertension or diabetes mellitus and PaCO_2._ AZT, azithromycin; PaO_2_, oxygen partial pressure, arterial; PaCO_2_, carbon dioxide partial pressure, arterial; FiO_2_, inspired fraction of oxygen; HCQ, hydroxychloroquine; ICU, intensive care unit.

**Table 2:** Characteristics of survivors and non-survivors at hospital discharge.

		Survivors	Non-Survivors	*p*-Value
Patients	N (%)	1022 (74.3)	354 (25.7)	
Age	years	68 (57–76)	77 (71–83)	<0.001
Body mass index	kg∗m^−2^	26 (24–29)	26 (24–29)	0.11
Male	N (%)	648 (63.4)	261 (73.7)	0.001
**Comorbidity**				
Arterial hypertension	N (%)	377 (36.9)	122 (34.5)	0.45
Diabetes mellitus	N (%)	204 (20.0)	85 (24.0)	0.12
**Laboratory data**				
PaO_2_	mmHg	57 (49–67)	47 (38–56)	<0.001
PaCO_2_	mmHg	34 (31–38)	33 (30–38)	0.003
PaO_2_/FiO_2_	mmHg	271 (231–314)	211 (162–254)	<0.001
pH		7.49 (7.46–7.51)	7.48 (7.43–7.51)	<0.001
Lactate	mmol∗L^−1^	1.0 (0.7–1.3)	1.3 (1.0–2.1)	<0.001
Leukocytes	10^9^∗L^−1^	7.2 (5.5–9.6)	7.6 (5.3–10.6)	0.15
Lymphocytes	10^9^∗L^−1^	1.0 (0.7–1.4)	0.8 (0.6–1.1)	<0.001
Platelets	10^9^∗L^−1^	185 (143–246)	164 (122–226)	<0.001
C-reactive protein	mg∗L^−1^	81 (35–153)	143 (91–198)	<0.001
Lactate dehydrogenase	U∗L^−1^	423 (32–616)	641 (472–795)	0.016
**Outcomes**				
ICU admission	N (%)	78 (7.6)	96 (27.1)	<0.001
Hospital length of stay	days	7 (5–11)	7 (3–11)	0.001

Demographics, comorbidities, laboratory data, and outcomes of survivors and non-survivors. Factor variables are expressed as count (%) and tested with chi-square test, continuous variables as median (IQR) and tested with Kruskal–Wallis test. PaO_2_, oxygen partial pressure, arterial; PaCO_2_, carbon dioxide partial pressure, arterial; FiO_2_, inspired fraction of oxygen; ICU, intensive care unit.

**Table 3:** Results of adjusted analysis.

	Model	AZT vs. Nneither Ttreatment	HCQ vs. Nneither Ttreatment	AZT and HCQ vs. Nneither Ttreatment
In-hospital mortality	Logit regression	0.60 (0.42–0.85)	0.76 (0.53–1.09)	1.13 (0.77–1.69)
ICU admission	Logit regression	1.08 (0.57–2.05)	1.10 (0.69–1.76)	1.82 (1.27–2.61)
Hospital length of stay	Poisson regression	1.17 (1.10–1.25)	1.15 (1.06–1.24)	1.34 (1.24–1.45)

Adjusted OR and 95% CI are reported for logit regression, adjusted incidence rate ratios (IRRs), and 95% CI for Poisson regression.

## Supplementary Materials

The following are available online at https://www.mdpi.com/2077-0383/9/9/2800/s1, Figure S1. Standardized mean difference between hydroxychloroquine and neither treatment before and after overlap weight propensity score. Figure S2. Standardized mean difference between azithromycin and neither treatment before and after overlap weight propensity score. Figure S3. Standardized mean difference between azithromycin and neither treatment before and after overlap weight propensity score. Table S1. Records of corticosteroids and/or enoxaparin use during hospital stay in the four groups of patients. Table S2. Analysis of completed cases. Table S3. Analysis excluding patients admitted to intensive care.
