# Supplementary material for: Impact of Azithromycin and/or Hydroxychloroquine on Hospital Mortality in COVID-19
Source: J Clin Med. 2020 Aug 30;9(9):2800. doi: 10.3390/jcm9092800 (PMC7563535; doi:10.3390/jcm9092800)
Supplement: Supplementary file 1 [file jcm-09-02800-s001.pdf]

Impact of Azithromycin and/or Hydroxychloroquine on Hospital Mortality in COVID-19

Filippo Albani[1] M.D., Federica Fusina[1] M.D., Alessia Giovannini[1] M.D., Pierluigi Ferretti[1] M.D., Anna Granato[1] M.D., Chiara Prezioso[1,2] M.D., Danilo Divizia[1] M.D., Alessandra Sabaini[1] M.D., Marco Marri[3] B.S., Elena Malpetti[1] M.D., Giuseppe Natalini[1] M.D.

Table of contents

Figure S1. Standardized mean difference between hydroxychloroquine and neither treatment before and after overlap weight propensity score.

Figure S2. Standardized mean difference between azithromycin and neither treatment before and after overlap weight propensity score.

Figure S3.Standardized mean difference between azithromycin and neither treatment before and after overlap weight propensity score.

Table S1. Records of corticosteroids and/or enoxaparin use during hospital stay in the four groups of patients.

Table S2.Analysis of completed cases

Table S3. Analysis excluding patients admitted to Intensive care.

Figure S1. Standardized mean difference between hydroxychloroquine and neither treatment before and after overlap weight propensity score.

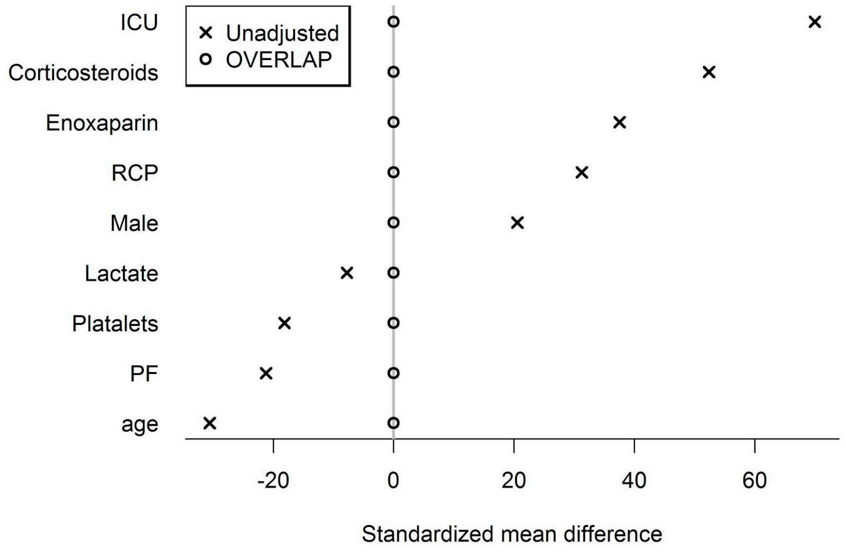

Admission to intensive care unit (ICU), sex male (Male), treatment during hospital admission with corticosteroids (Corticosteroids) and/or Enoxaparin (Enoxaparin). Laboratory data day of admission: serum lactate (Lactate), platelets count (Platelets), C-reactive protein (CRP), PaO_2/FiO_2 (PF).

Figure S2. Standardized mean difference between azithromycin and neither treatment before and after overlap weight propensity score.

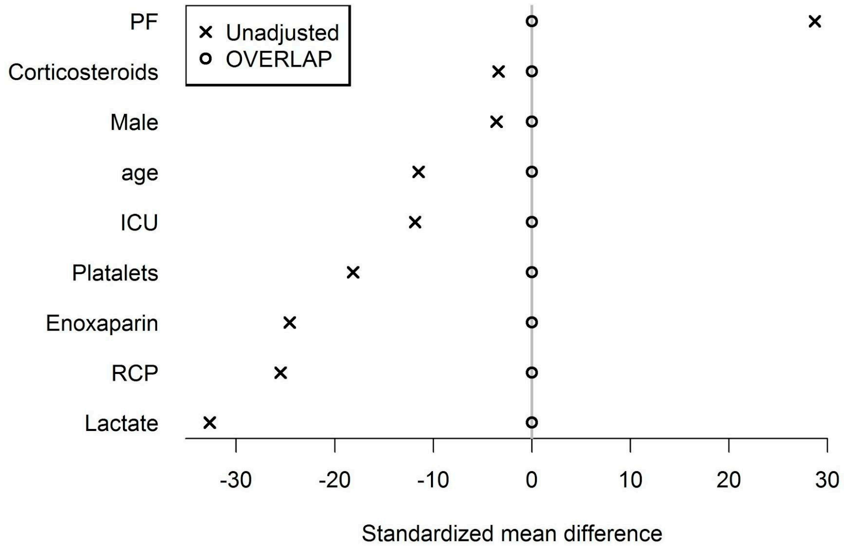

Admission to intensive care unit (ICU), sex male (Male), treatment during hospital admission with corticosteroids (Corticosteroids) and/or Enoxaparin (Enoxaparin). Laboratory data day of admission: serum lactate (Lactate), platelets count (Platelets), C-reactive protein (CRP), PaO_2/FiO_2(PF).

Figure S3.Standardized mean difference between azithromycin and neither treatment before and after overlap weight propensity score.

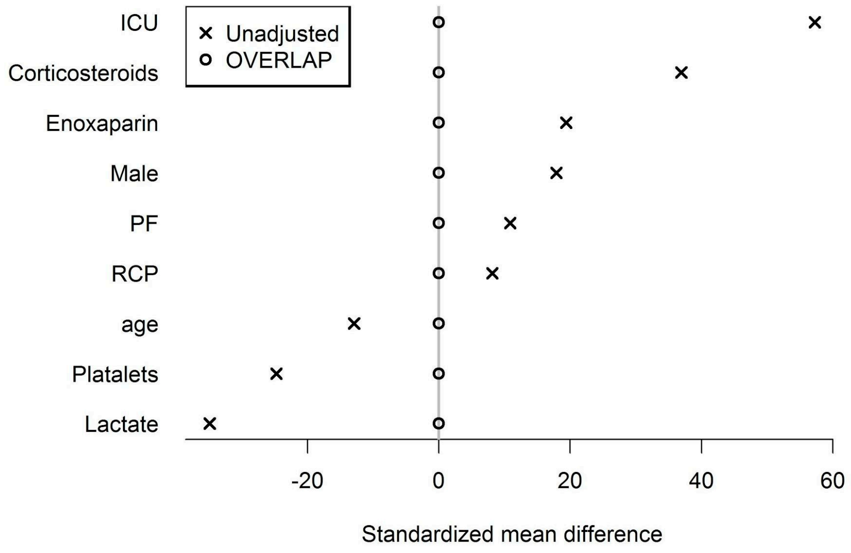

Admission to intensive care unit (ICU), sex male (Male), treatment during hospital admission with corticosteroids (Corticosteroids) and\or Enoxaparin (Enoxaparin). Laboratory data day of admission: serum lactate (Lactate), platelets count (Platelets), C-reactive protein (CRP), PaO_2/FiO_2(PF).

Table S1. Records of corticosteroids and/or enoxaparin use during hospital stay in the four groups of patients.

	Neither treatment	HCQ alone	AZT alone	AZT and HCQ	*p*-Value
Enoxaparin	344 (56.9)	157 (74.4)	188 (44.7)	110 (66.3)	<0.001
Corticosteroids	208 (34.4)	126 (59.7)	138 (32.8)	87 (52.4)	<0.001

Data were recorded if patients were treated with at least one dose at any time during hospital stay; variables are expressed as count (%). Hydroxychloroquine (HCQ); Azithromycin (AZT).

Table S2.Analysis of completed cases.

	Model	AZT vs. Neither treatment	HCQ vs. Neither treatment	AZT and HCQ vs. Neither treatment
In-hospital mortality	Logit regression	0.59 (0.40-0.89)	0.89 (0.59-1.34)	0.94 (0.60-1.47)
Admission ICU	Logit regression	0.79 (0.37-1.69)	1.11 (0.69-1.80)	1.43 (0.94-2.19)
Hospital length of stay	Poisson regression	1.25 (1.16-1.34)	1.13 (1.04-1.23)	1.38 (1.26-1.50)

Odds ratios (ORs) and 95% confidence intervals are reported for logit regression, incidence rate ratios (IRRs) and 95% confidence intervals for Poisson regression. Hydroxychloroquine (HCQ); Azithromycin (AZT).

Table S3. Analysis excluding patients admitted to intensive care.

	Model	AZT vs. Neither treatment	HCQ vs. Neither treatment	AZT and HCQ vs. Neither treatment
In-hospital mortality	Logit regression	0.53 (0.36-0.78)	0.80 (0.51-1.25)	1.00 (0.61-1.65)
Hospital length of stay	Poisson regression	1.19 (1.11-1.28)	1.28 (1.16-1.41)	1.52 (1.38-1.68)

Odds ratios (ORs) and 95% confidence intervals are reported for logit regression, incidence rate ratios (IRRs) and 95% confidence intervals for Poisson regression. Hydroxychloroquine (HCQ); Azithromycin (AZT).
